# Capsular warning syndrome—a case of atrial fibrillation and corona radiata infarct

**DOI:** 10.1186/s12245-023-00541-w

**Published:** 2023-09-26

**Authors:** Sarah En Mei Tan, Kenneth Wei Jian Heng

**Affiliations:** Department of Emergency Medicine, Tan Tock Seng Hospital, 11 Jalan Tan Tock Seng, Singapore, 308433 Singapore

**Keywords:** Transient ischaemic attack, Capsular warning syndrome, Anti-platelet therapy, Stroke, Infarct, Atrial fibrillation, Emergency department, Neurology

## Abstract

**Background:**

Capsular warning syndrome (CWS) is a rare clinical syndrome characterised by recurrent and transient episodes of focal neurological deficits with high risk of infarction. The exact physiological mechanism of CWS remains unclear but is most commonly believed to be a result of haemodynamic insufficiency in diseased, small penetrating vessels. There are no defined treatment guidelines or established effective therapy.

**Case presentation:**

We describe the case of a 65-year-old man who presented to the emergency department with recurrent episodes of dysarthria coupled with right facial droop and right-sided weakness. Symptoms recurred a total of ten times within a span of 3 h. He had new onset atrial fibrillation. An initial cerebral angiogram showed mild intracranial atherosclerotic disease with no proximal large vessel occlusion or acute infarct. Magnetic resonance imaging 1 h later demonstrated an infarct in the left corona radiata.

**Conclusions:**

This case illustrates an uncommon etiology of CWS. We will also discuss the lack of consensus in treatment options for CWS to mitigate a complete stroke.

## Background

The entity capsular warning syndrome (CWS) was first coined by Donnan et al. in 1993 to describe the phenomenon of at least 3 crescendo transient ischaemic attacks (TIAs) restricted to the region of the internal capsule, usually causing symptoms affecting the face, arm, and leg within a 24-h period [1]. Later studies used broader timeframes to define CWS up to 7 days, but it remains rare and constitutes only 1.5 to 4.5% of TIAs [2]. CWS has been associated with the highest stroke risk of up to 30% out of all TIA types [3]. As many as 71% of CWS patients eventually develop permanent infarction, yet little has been published about its prognosis, management strategies, and treatment outcomes [4]. The mechanism of CWS remains unclear, and most attribute the pathogenesis to atherosclerosis of small penetrating vessels [5]. Multiple treatments such as blood pressure control [6], anti-platelet or anti-coagulant therapy [7], and thrombolysis [8] have been suggested, but optimal clinical management remains controversial. Our case illustrates a cardioembolic cause of CWS and highlights how it can be challenging to manage on initial presentation to the emergency department (ED) to mitigate possible disease progression into a complete stroke.

## Case presentation

**Fig. 1:**
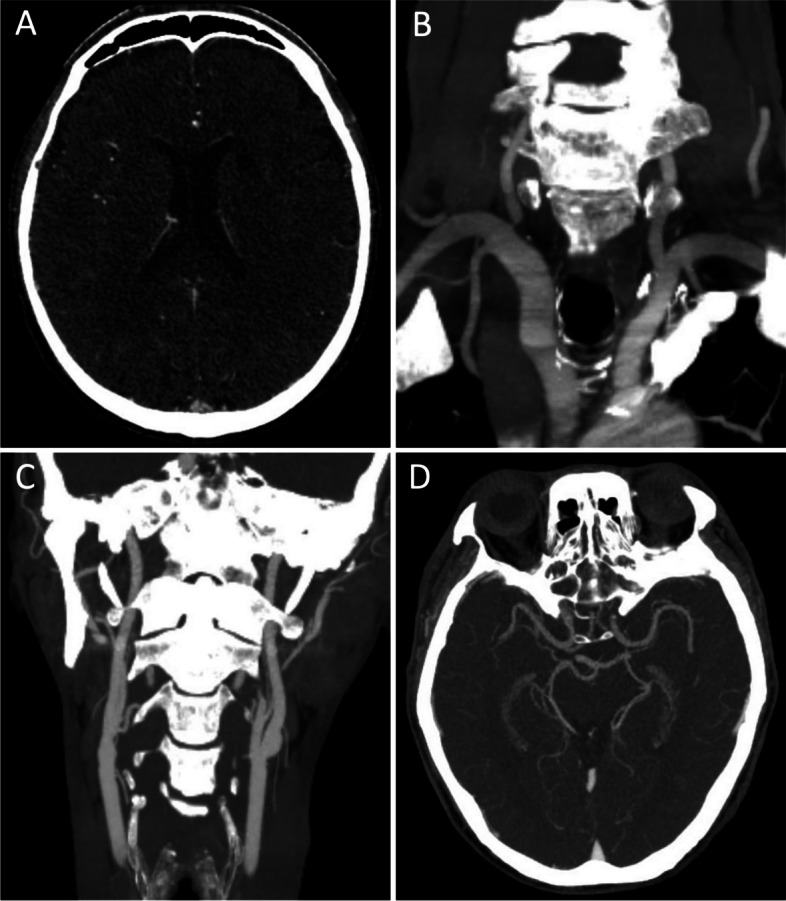
Computed tomography angiogram brain. **A** Chronic lacunar infarct in left striatocapsular region. **B** Focal mixed plaque at origin of right vertebral artery causing severe stenosis with preserved distal opacification. **C** No large vessel occlusion of major cervical vessels. **D** No major artery occlusion or stenosis in anterior and posterior circulations

**Fig. 2:**
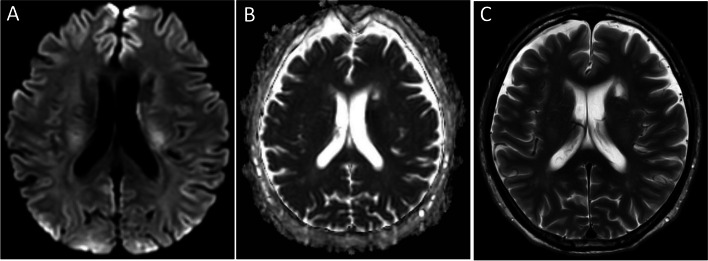
Magnetic resonance imaging brain. **A** Diffusion-weighted imaging (DWI). **B** Apparent diffusion co-efficient (ADC). **C** T2 weighted (T2W). Restricted diffusion in left corona radiata periventricular region on DWI and ADC with no associated T2W hyperintensity, suggestive of an acute infarct

A previously well 65-year-old man with no past medical or smoking history of note presented to our ED with slurring of speech and right-sided weakness that started 1 h prior. Symptoms were noted by the paramedics to have resolved completely enroute in the ambulance but recurred on reaching the ED. On arrival, his blood pressure was 156/127 mmHg and heart rate 106 beats/minute. Neurological examination revealed dysarthria, right cranial nerve VII upper motor neuron palsy, and right upper and lower limb weakness, constituting a National Institutes of Health Stroke Scale (NIHSS) score of 9. Deficits resolved completely 10 min into consultation only to recur again 5 min later. Cardiac monitoring revealed atrial fibrillation with a rapid ventricular rhythm and varying heart rate from 90 to 144 beats/minute. Cardiorespiratory examination was unremarkable. Brain computed tomography angiogram (CTA) depicted an old lacunar infarct in the left striatocapsular region and mild atherosclerotic disease of the cervical and intracranial major arteries with no proximal large vessel occlusion (Fig. 1). The Alberta Stroke Program Early CT Score (ASPECTS) was 10. The patient had a total of five episodes of transient deficits over the same areas within a span of 2 h from his symptom-discovery time but was again symptom-free at the conclusion of the CTA. He was started on intravenous hydration and loaded aspirin 300 mg, clopidogrel 300 mg, and atorvastatin 80 mg. The patient continued to develop recurrences of symptoms post-CTA another five times with each episode lasting less than 10 min. A brain magnetic resonance imaging (MRI) and magnetic resonance angiography (MRA) performed 3 h from initial symptom onset revealed a small acute non-haemorrhagic infarct in the left corona radiata periventricular region with no large vessel occlusion or severe stenosis (Fig. 2). The patient was admitted to the Neurology High Dependency Unit for close monitoring with an NIHSS score of 0.

During the patient’s hospitalisation, he was maintained on aggressive intravenous hydration and continued to be free from neurological deficits. Serum total cholesterol and low-density lipoprotein were elevated at 7.3 mmol/L and 5.6 mmol/L respectively. Glycosylated haemoglobin was normal. Blood coagulation studies showed normal prothrombin time, activated partial thromboplastin time, and international normalised ratio. Telemetry monitoring confirmed new-onset atrial fibrillation. Extended electrolytes, free thyroxine, and troponin were unremarkable. Blood pressure remained less than 130/80 mmHg and heart rate less than 90 beats/minute without pharmacological therapy. Dual anti-platelet agents were ceased and replaced with apixaban 5 mg twice a day on day 3 of admission. The patient was discharged without residual deficit on day 5 of admission with apixaban 5 mg twice a day and high dose atorvastatin for 2 weeks followed by 40 mg every morning.

## Discussion and conclusions

This case describes an unusual cardioembolic cause of CWS. The exact pathophysiological mechanism of CWS remains unclear. Diabetes, hypertension, hyperlipidemia, and smoking have been correlated to CWS, and most studies suggest atherosclerosis of small penetrating vessels as the likely underlying pathology [3, 9]. When Donnan et al. first described CWS, he specified no evidence of thrombo- or cardioembolic phenomena when cerebral infarction did develop [1]. As the CTA does show mild atherosclerotic disease, one can perhaps argue this is a case of small vessel CWS, and the atrial fibrillation might merely be a pure coincidence or a result of stroke-induced sympathetic stimulation. A series of transesophageal echocardiograms performed on patients with ischaemic strokes did illustrate 20% of those with lacunar infarcts actually had a cardiac cause with major risk factors for embolization [10]. Though we are unable to definitively prove the etiology in this case, the new-onset atrial fibrillation demonstrates at least an association and possibly a causational relationship where a cardiac embolus might have travelled into a lenticulostriate artery.

As the mechanism of CWS remains unclear, there is also uncertainty as to whether proposed therapies alter the natural progression of the disease. Different treatment strategies have been considered, including thrombolysis, anti-coagulation, dual anti-platelet agents, elevating blood pressure, and aggressive intravenous hydration [11]. Existing evidence published thus far is mostly limited to observational studies and case series. Functional prognosis is depicted to be favourable in most patients with CWS, but the 7-day stroke risk can reach as high as 60% [12].

Studies have shown the mean duration of recurrent TIA episodes in CWS to vary from 6 to 24 min, and the internal capsule remains the most frequently involved location of established infarct on MRI at 50 to 70% [11]. Our patient had multiple episodes each lasting less than 10 min and fortunately did not suffer residual functional deficits despite MRI depicting a left corona radiata infarct. He was initially loaded with dual anti-platelet agents, and there was a standing order by the consulting neurologist to offer intravenous recombinant tissue plasminogen activator (rTPA) should an episode persist beyond 30 min. Current rTPA trials based their decisions on CT and clinical findings, and in many cases, MRI findings are uncertain. The definition of CWS should therefore include patients with TIA and those with transient symptoms of infarction because most of them were diagnosed clinically without the benefit of MRI before treatment [13]. A post hoc analysis of the WAKE-UP trial did show similar favourable functional outcomes between patients with lacunar infarcts and other stroke subtypes who were treated with rTPA, suggesting it could possibly be an effective treatment measure for CWS though more studies will need to be done to ascertain this [14]. Thus far, limited studies have shown no statistically significant difference in therapeutic effects or functional outcome amongst CWS patients treated with rTPA or anti-platelet agents [3, 15, 16]. There is also no strong evidence for the efficacy of anti-coagulant therapy in the acute phase of CWS. Some case studies have shown benefit of double anti-platelet therapy over single therapy as a secondary prevention strategy [17, 18].

In conclusion, this case demonstrates that CWS can be caused by cardioembolic phenomena in the setting of atrial fibrillation. The rarity of this entity has limited research on optimal management, but dual anti-platelet agents have been safely used in non-cardioembolic CWS and appear to be a viable treatment in cardioembolic CWS patients prior to transitioning to anti-coagulants. There needs to be more research or randomised controlled trials focused on treatment options that can alter the disease progression of CWS, but this would be difficult considering the paucity of cases.

## Data Availability

Not applicable.

## Abbreviations

CWS: Capsular warning syndrome
ED: Emergency department
TIA: Transient ischaemic attack
NIHSS: National Institutes of Health Stroke Scale
CTA: Computed tomography angiogram
ASPECTS: Alberta Stroke Program Early CT Score
MRI: Magnetic resonance imaging
MRA: Magnetic resonance angiography
rTPA: Recombinant tissue plasminogen activator
